# Bendable Single Crystal Silicon Nanomembrane Thin Film Transistors with Improved Low-Temperature Processed Metal/n-Si Ohmic Contact by Inserting TiO_2_ Interlayer

**DOI:** 10.3390/nano8121060

**Published:** 2018-12-16

**Authors:** Jiaqi Zhang, Yi Zhang, Dazheng Chen, Weidong Zhu, He Xi, Jincheng Zhang, Chunfu Zhang, Yue Hao

**Affiliations:** State Key Discipline Laboratory of Wide Band Gap Semiconductor Technology, School of Microelectronics, Xidian University, 2 South Taibai Road, Xi’an 710071, China; 18031362893@163.com (J.Z.); hyper_sys@163.com (Y.Z.); dzchen@xidian.edu.cn (D.C.), wdzhu@xidian.edu.cn (W.Z.); hxi@xidian.edu.cn (H.X.); jchzhang@xidian.edu.cn (J.Z.); yhao@xidian.edu.cn (Y.H.)

**Keywords:** thin film transistor, single-crystal Si nanomembrane (Si NMs), TiO_2_ insertion layer, ohmic contact

## Abstract

Bendable single crystal silicon nanomembrane thin film transistors (SiNMs TFTs), employing a simple method which can improve the metal/n-Silicon (Si) contact characteristics by inserting the titanium dioxide (TiO_2_) interlayer deposited by atomic layer deposition (ALD) at a low temperature (90 °C), are fabricated on ITO/PET flexible substrates. Current-voltage characteristics of titanium (Ti)/insertion layer (IL)/n-Si structures demonstrates that they are typically ohmic contacts. X-ray photoelectron spectroscopy (XPS) results determines that TiO_2_ is oxygen-vacancies rich, which may dope TiO_2_ and contribute to a lower resistance. By inserting TiO_2_ between Ti and n-Si, I_ds_ of bendable single crystal SiNMs TFTs increases 3–10 times than those without the TiO_2_ insertion layer. The fabricated bendable devices show superior flexible properties. The TFTs, whose electrical properties keeps almost unchanged in 800 cycles bending with a bending radius of 0.75 cm, obtains the durability in bending test. All of the results confirm that it is a promising method to insert the TiO_2_ interlayer for improving the Metal/n-Si ohmic contact in fabrication of bendable single crystal SiNMs TFTs.

## 1. Introduction

Flexible electronics is an important development direction in the field of future electronics. Scientists can use flexible materials to fabricate advanced electronic devices, such as transistor arrays for optional folding and stretching, bendable flexible screens, or some sensors which can be integrated on the clothing [1,2,3,4,5,6,7,8,9]. Advances in various flexible electronic technologies, including solar cells, sensors and displays, have been driven by the use of flexible organic materials. However, organic-based semiconductors suffer from poor device performance due to their low carrier mobility and their chemical/thermal instability. Recently, the discovery of single-crystal Si nanomembranes (SiNMs) has fascinated the flexible electronics community because of their high carrier mobility, stable chemical/thermal properties and flexibility. Particularly, SiNMs released from silicon-on-insulator (SOI) become one of the best choices owing to their outstanding electrical properties, mature fabrication techniques and commercial feasibility at relatively lower cost [10,11,12,13,14].

For SiNMs, effectively doping them to form effective ohmic contacts and realizing low contact resistivity are both critical in realizing high speed operation. Nevertheless, many flexible substrates are soft and have very low processing temperature tolerance. For example, ITO/PET substrates just can withstand the highest temperature as low as 150 °C [11]. Hence, the traditional high-temperature processing cannot be directly used. This challenge has been partially overcome by employing a pre-doped (ion implantation and annealing before SiNMs release) SiNMs transfer and gate-last TFTs fabrication process [10,11]. However, a low-temperature process to achieve a lower Metal/Si Ohmic contact is still urgently pursued.

On the basis of the works above, we reports a simple method that using ALD technology deposits TiO_2_ at a low temperature of 90 °C to further improve the contact between the source/drain regions and metal electrodes. Current-voltage characteristics of Ti/insertion layer (IL)/n-Si structures, XPS results of TiO_2_ and the normalized current-voltage characteristics of bendable single crystal silicon TFTs with different cycles of TiO_2_ are obtained and described in detail. By inserting titanium dioxide, good ohmic contacts are formed between the source/drain regions and metal electrodes. I_ds_ of bendable single crystal SiNMs TFTs increases 3–10 times than those without the TiO_2_ insertion layer. The TFTs, whose electrical properties keeps almost unchanged in 800 cycles bending with a bending radius of 0.75 cm, obtains the durability in bending test.

## 2. Materials and Methods

### 2.1. Device Fabrication

**Figure 1:**
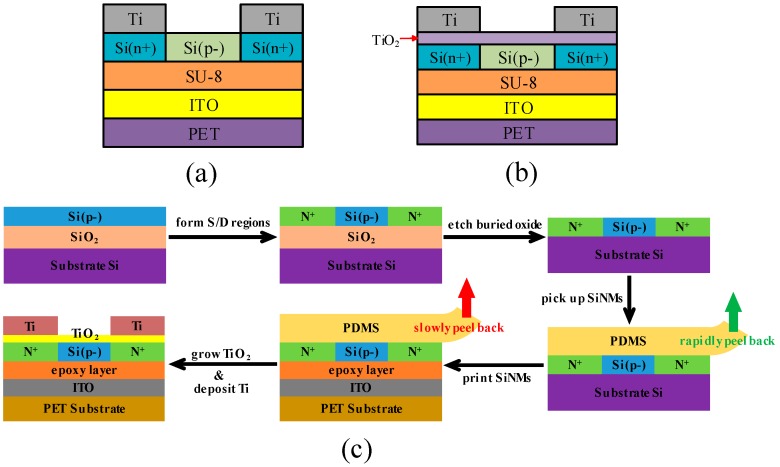
(**a**) Structure schematic of a bendable single crystal silicon TFT without inserting TiO_2_. (**b**) Structure schematic of a bendable single crystal silicon TFT with inserting different cycles of TiO_2_. (**c**) Schematic illustration of fabrication process for bendable single crystal silicon TFT.

Figure 1 schematically illustrates the cross section of the two kinds of bendable single crystal silicon TFTs built on ITO/PET substrates and the devices fabrication process. Figure 1a shows structure schematic of a bendable single crystal silicon TFT without inserting TiO_2_ and Figure 1b shows structure schematic of a bendable single crystal silicon TFT with inserting different cycles of TiO_2_. Figure 1c shows that the devices fabrication process was started with silicon-on-insulator wafer (SOI) (Soitec by Smartcut with 200 nm top Si which is doped boron whose level is 1 × 10^14^ cm^−3^ and 200 nm buried oxide). In our process of making n-channel TFTs, the source/drain regions were first formed on the SOI substrate via phosphorus ion implantation with a dose of 5 × 10^15^ cm^−2^ and an energy of 30 keV followed by annealing in RTP at 1000 °C for 20 s in N_2_ ambient to activate the implanted dopants. The doping concentration of the contact area is ~10^19^ cm^−3^. SOI wafer was patterned by lithography to form the hole patterns. RIE was used to etch the holes. 33% hydrofluoric acid (HF) etched buried oxide (SiO_2_) through a lot of etching holes above SiO_2_ [15,16,17,18,19]. Completely etching buried oxide took about 48 h so that the top Si dropped on the bottom silicon substrate by Van der Waals force [20,21,22]. Deionized water was used to wash away residual HF to be ready for transfer. In order to avoid being dislocated and scattered of the SiNMs which was immersed in HF, we designed the mask including a lot of 1 × 1 cm units so that the SiNMs formed a very large area. Even if the buried oxygen was completely etched off, there was no displacement and scatter of the SiNMs on the bottom Si. At the same time, this design increased the transfer area reaching 1 cm^2^. A flat piece of polydimethylsiloxane (PDMS) was brought into conformal contact with the top surface of the wafer and then rapidly peeled back to pick up SiNMs. The interaction between SiNMs and PDMS is sufficient to pick up SiNMs with good efficiency, nearly 100%. A flexible ITO/PET substrate served as the target substrate. The target substrate was washed with acetone and alcohol, rinsed with deionized water and then dried with a stream of nitrogen. Treating the ITO/PET substrate with a short O_2_ plasma (20 sccm O_2_ flow with 50 W rf power for 10 s) promoted adhesion between it and a spin coating dielectric layer of epoxy (4000 rpm for 30 s of SU8-2002). Then the SiNMs on PDMS was brought against the epoxy layer (SU8-2002). SiNMs was transferred to the ITO/PET substrate by gently pressing and slowly peeling up PDMS. The epoxy layer was cured at 100 °C for 1 min, exposed to UV light from the backside of the sample for 10 s and finally post-baked at 100 °C for 1 min. Photolithography defined a pattern on the substrate. RIE etched the Si to form MESA. O_2_ plasma was used to remove photoresist. After 5% HF solution immersion for 2 min and de-ionized water rinse for 2 min, N_2_ gun blew dry the samples. Then the samples were immediately loaded into PicosunTM R-200 Advanced ALD chamber. Titanium tetrakis (dimethylamide) (TDMATi) was used as Ti source and H_2_O was used as oxygen source. During the process, TDMATi source bottle was heated to 120 °C and the N_2_ carrier flow was set to 15 standard cubic centimeter per minute (sccm), pulse time and purge time were 100 ms and 40 s respectively. For oxygen source, the N_2_ carrier flow was set to 15 sccm, pulse time was 100 ms and purge time was 40 s. The whole deposition process was carried out at 90 °C. There were two different cycles of 5 and 10. Using these two conditions formed ~0.5 and ~1 nm TiO_2_ on the SiNMs, respectively. Photolithography defined a pattern on the samples. Put them into a PRO LINE PVD 75 SYSTEM (Kurt J. Lesker Company, Pittsburgh, PA, USA) to deposit Ti (100 nm) by electron beam evaporation. Finally, the source and drain metal contacts formed on the low-resistive source and drain regions followed by lift-off without any further thermal treatment.

### 2.2. Device Characterization

Etching depth of SiNMs was measured by Stylus Profiler (Bruker Dektak XT, Bremen, Germany). The XPS testing of TiO_2_ samples was performed on Thermo escalab 250Xi (Thermo Fisher Scientific, Waltham, MA, USA) using monochromatic Al-Ka (1486.6 eV) as the radiation source. The I–V characteristics of Ti/insertion layer (IL)/n-Si metal-insulator-semiconductor (MIS) structures and bendable single crystal silicon TFTs were both measured by using Keithley 1500 semiconductor characterization system (Tektronix, Inc., Beaverton, OR, USA). All the measurements were performed under ambient atmosphere at room temperature without encapsulation.

## 3. Results and Discussion

**Figure 2:**
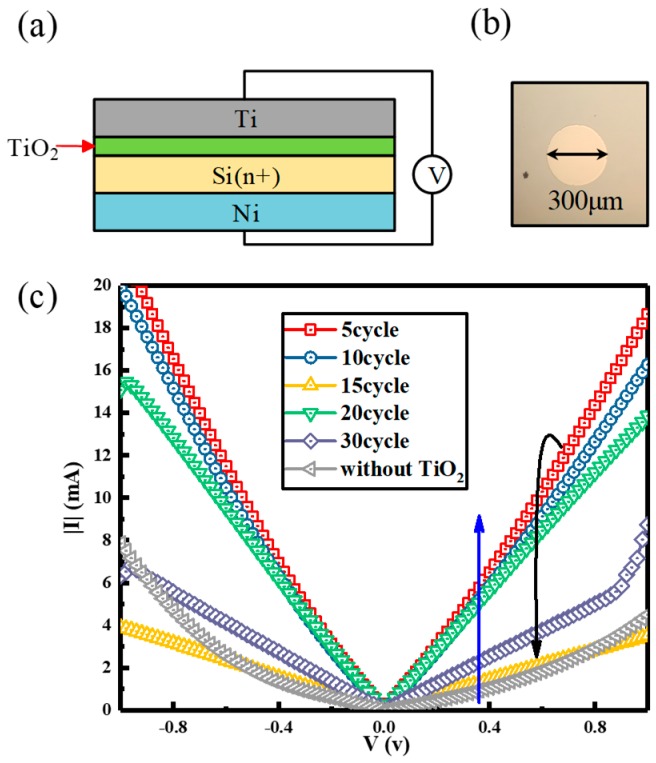
(**a**) Schematic cross-sectional view of the Ti/insertion layer (IL)/n-Si metal-insulator-semiconductor (MIS) structures with different ALD cycles. (**b**) A high magnification optical images of the MIS structure whose Ti pad diameter is 300 μm. (**c**) The I–V characteristics of Ti/insertion layer (IL)/n-Si (doping level is ~10^19^ cm^−3^) MIS structure with different ALD cycles.

Figure 2a presents a schematic cross-sectional view of the Ti/insertion layer (IL)/n-Si metal-insulator-semiconductor (MIS) structures with different ALD cycles. Figure 2b shows a high magnification optical images of the MIS structure whose Ti pad diameter is 300 μm. Figure 2c shows the I–V characteristics of Ti/insertion layer (IL)/n-Si (doping level is ~10^19^ cm^−3^) MIS structure with different ALD cycles. It is obvious that the characteristic curve of devices without TiO_2_ (0 cycle) is curving which is a typical Schottky contact. It indicates that even if the source/drain area is heavily doped, Ti/n-Si (doping level is ~10^19^ cm^−3^) will not form a good ohmic contact without any treatment. Amazingly, the characteristic curves of devices which are deposited by the TiO_2_ insertion layer are all typically good ohmic contacts. The contact resistance of 5 cycle is the smallest and the contact resistance of 10 cycle is the second smallest. However, with the increase of the cycle (the thickness of TiO_2_), the contact resistance becomes greater and greater. The explanation for this is that the thickness of titanium dioxide exceeds a certain value leading to carriers passing through the insertion layer at lower tunneling rate [23,24]. This will reduce the current, with presenting a larger contact resistance. Therefore, we chose 5 and 10 cycles of titanium dioxide inserting into the TFTs to compare those without titanium dioxide insertion layer.

**Figure 3:**
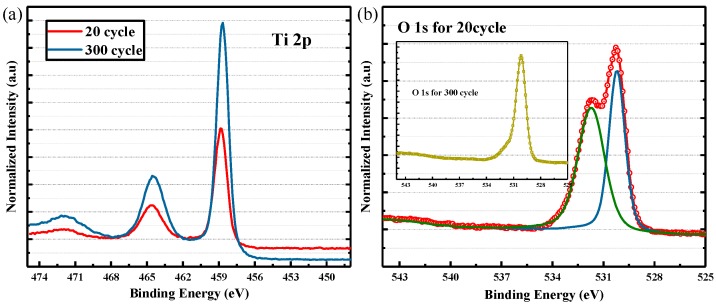
(**a**) The XPS results of Ti 2p of samples for 20 cycles and 300 cycles of ALD process. (**b**) The XPS results of O 1 s with thin TiO_2_, shoulder left to O 1 s peak is obvious. The illustration in the upper left corner shows the fitting curve of 300 cycles.

Figure 3a shows the XPS results of Ti 2p of samples for 20 cycles and 300 cycles of ALD process and all the XPS results are calibrated with C 1 s peak at 284.8 eV [23,24]. The two curves have two distinct peaks at about 458–459 eV and 464–465 eV, which represent for Ti 2p3/2 and Ti 2p1/2 peaks and are consistent with typical values of TiO_2_. Figure 3b shows that O 1 s results with thin TiO_2_, shoulder left to O 1 s peak is obvious. This shoulder located at 531.5 eV represents for oxygen vacancies and it indicates that thin sample has more oxygen vacancies. The illustration in the upper left corner shows the fitting curve of 300 cycles. It can be seen that the peak of oxygen vacancies is not obvious. In conclusion, there are some oxygen vacancies in thin TiO_2_ film, which will dope TiO_2_ and make it more conductive [24]. Due to the presence of a donor band related to oxygen vacancies which can provide more electrons, TiO_2_ behaves as an n-type semiconductor and exhibits good electrical conductivity.

**Figure 4:**
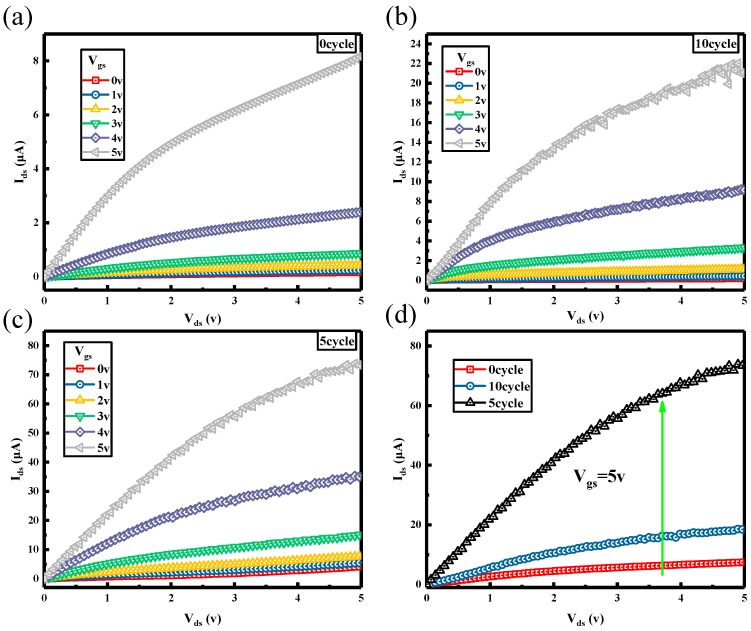
The normalized current-voltage characteristics of the thin film transistor devices on PET substrate with different cycle of TiO_2_. (**a**) The thin film transistor devices on PET substrate with 0 cycle of TiO_2_. (**b**) The thin film transistor devices on PET substrate with 10 cycles of TiO_2_. (**c**) The thin film transistor devices on PET substrate with 5 cycles of TiO_2_. (**d**) Shows the normalized current-voltage characteristics of the thin film transistor devices on PET substrate whose I_ds_ of 0, 5, 10 cycles at V_gs_ = 5 v.

Figure 4a–d presents the normalized current-voltage characteristics of TFTs with different cycles of TiO_2_. As shown in the figure, the I_ds_ of 0 cycle is the smallest. The I_ds_ of 5 cycle is the greatest. Figure 4d shows the I_ds_ of 0, 5, 10 cycle at V_gs_ = 5 v. It presents that the current of 10 cycles is three times the 0 cycle one. The current of 5 cycles is ten times the 0 cycle one. These results are consistent with results of Figure 2c. The reason is that inserting the titanium dioxide of appropriate thickness between Ti and n-Si can effectively restrain the Fermi energy level pinning effect of n-type silicon so as to improve the Ti/n-Si ohmic contact, reduce the contact resistance and increase the current driving ability.

**Figure 5:**
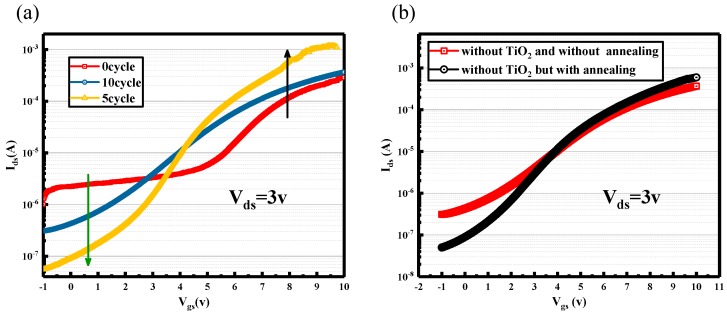
(**a**) Transfer characteristics of TFTs on ITO/PET substrates with different cycles of TiO_2_. (**b**) Transfer characteristics of the annealed TFT which are not deposited TiO_2_ on ITO/PET substrates compared with one without annealing.

Figure 5a presents transfer characteristics of TFTs with different cycles of TiO_2_. I_on/off_ of 0 cycle is 10^2^, 5 cycles is 10^4^ and 10 cycles is 10^3^. I_on_ of 0 cycle is smallest, 5 cycles is largest and 10 cycles is medium. This result is consistent with results of Figure 2c and Figure 4d. Thin TiO_2_ which restrains the Fermi energy level pinning effect of n-Si can improve the interface contact between Ti/n-Si contributing to increase I_ds_ [23,24,25]. I_off_ of 0 cycle is largest, 5 cycles is smallest and 10 cycles is medium. For transistors, the smaller the I_off_, the better the performance of the device. After analysis, it may be that ALD growth of titanium oxide plays anneal role so that the interface contact between epoxy layer (SU8-2002) and SiNMs is better than that without depositing TiO_2_. It promotes the ability to control current of gate electrode. To test this conjecture, we put the TFT without depositing TiO_2_ into ALD annealing at 90 °C for 5 min (the time of depositing 5 cycles TiO_2_) and then compared the transfer characteristics with unannealed one. Results are as Figure 5b shown, the I_off_ of without TiO_2_ but with annealing is an order of magnitude smaller than that without TiO_2_ and without annealing, which is consistent with results of Figure 5a. Hence, it indicates that even if annealing at 90 °C can also improve the interface contact between epoxy layer (SU8-2002) and SiNMs with reducing interface defects and enhancing the ability to control current of gate electrode to reduce I_off_. Thus, the combination of the inserted TiO_2_ layer and very low-temperature annealing process improves the metal/Si ohmic contact.

**Figure 6:**
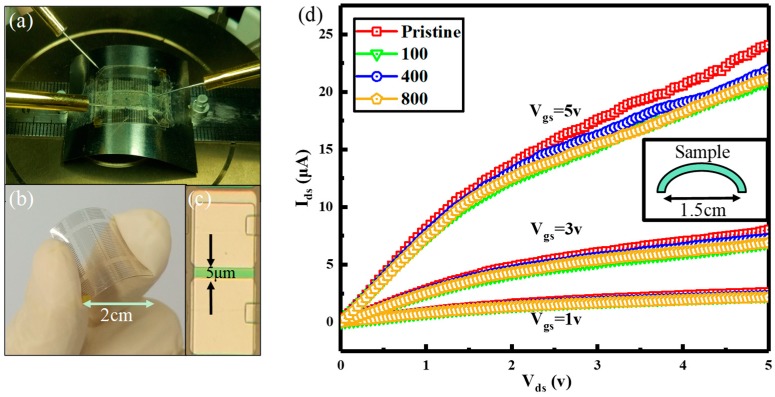
(**a**) The image of the flexible TFTs fixed on a probe station with the bending radius of 0.75 cm. (**b**) Shows the digital photographs of the flexible TFTs. (**c**) Shows an image of one device of TFTs under an optical microscope. (**d**) Shows the electrical characteristics of the flexible TFTs with a bending radius of 0.75 cm after being bent several times.

Figure 6a is an image of the flexible TFTs fixed on a probe station with the bending radius of 0.75 cm. Figure 6b shows the digital photographs of the flexible TFTs. Figure 6c shows an image of one device of TFTs under an optical microscope. Figure 6d shows a bending test of the flexible TFTs with a bending radius of 0.75 cm. Obtaining the durability in bending conditions is essential to wearable applications [26]. The electrical properties of the devices do not change significantly in 800 cycles with a bending radius of 0.75 cm, confirming a reliable performance for flexible operations.

## 4. Conclusions

A simple method was used to improve Ti/n-Si contact characteristics by inserting ALD deposited TiO_2_. The fabrication temperature of inserting ALD deposited TiO_2_ is below 120 °C which is the highest temperature plastic can withstand. Hence, we were able to combine this method into the manufacture of flexible thin film transistors. The results show that this method is not only feasible but also effective. It can change the Schottky contact into a satisfactory ohmic contact so that increase current drive capability. The electrical properties of the flexible TFTs do not change significantly with bending 800 cycles, confirming a reliable performance for flexible operations.

## References

[1] Kim J., Lee M., Shim H.J., Ghaffari R., Cho H.R., Son D., Jung Y.H., Soh M., Choi C., Jung S. (2014). Stretchable silicon nanoribbon electronics for skin prosthesis. Nat. Commun..

[2] Ying M., Bonifas A.P., Lu N., Su Y., Li R., Cheng H., Ameen A., Huang Y., Rogers J.A. (2012). Silicon nanomembranes for fingertip electronics. Nanotechnology.

[3] Park M., Kim M.S., Park Y.K., Ahn J.H. (2015). Si membrane based tactile sensor with active matrix circuitry for artificial skin applications. Appl. Phys. Lett..

[4] Won S.M., Kim H.S., Lu N., Kim D.G., Del Solar C., Duenas T., Ameen A., Rogers J.A. (2011). Piezoresistive strain sensors and multiplexed arrays using assemblies of single-crystalline silicon nanoribbons on plastic substrates. IEEE Trans. Electron Devices.

[5] Ahn J.H., Kim H.S., Lee K.J., Jeon S., Kang S.J., Sun Y., Nuzzo R.G., Rogers J.A. (2006). Heterogeneous three-dimensional electronics by use of printed semiconductor nanomaterials. Science.

[6] Yang S., Lu N. (2013). Gauge factor and stretchability of silicon-on-polymer strain gauges. Sensors.

[7] Lu N., Kim D.-H. (2014). Flexible and Stretchable Electronics Paving the Way for Soft Robotics. Soft Robot..

[8] Kim D.H., Ahn J.H., Kim H.S., Lee K.J., Kim T.H., Yu C.J., Nuzzo R.G., Rogers J.A. (2008). Complementary logic gates and ring oscillators on plastic substrates by use of printed ribbons of single-crystalline silicon. IEEE Electron Device Lett..

[9] Ahn J.H., Kim H.S., Menard E., Lee K.J., Zhu Z., Kim D.H., Nuzzo R.G., Rogers J.A., Amlani I., Kushner V. (2007). Bendable integrated circuits on plastic substrates by use of printed ribbons of single-crystalline silicon. Appl. Phys. Lett..

[10] Sun L., Qin G., Seo J.H., Celler G.K., Zhou W., Ma Z. (2010). 12-GHz thin-film transistors on transferrable silicon nanomembranes for high-performance flexible electronics. Small.

[11] Yuan H.C., Celler G.K., Ma Z. (2007). 7.8-GHz flexible thin-film transistors on a low-temperature plastic substrate. J. Appl. Phys..

[12] Torres Sevilla G.A., Almuslem A.S., Gumus A., Hussain A.M., Cruz M.E., Hussain M.M. (2016). High performance high-κ/metal gate complementary metal oxide semiconductor circuit element on flexible silicon. Appl. Phys. Lett..

[13] Gupta S., Navaraj W.T., Lorenzelli L., Dahiya R. (2018). Ultra-thin chips for high-performance flexible electronics. npj Flex. Electron..

[14] Zhang K., Seo J.H., Zhou W., Ma Z. (2012). Fast flexible electronics using transferrable silicon nanomembranes. J. Physics D Appl. Phys..

[15] Cohen G.M., Mooney P.M., Paruchuri V.K., Hovel H.J. (2005). Dislocation-free strained silicon-on-silicon by in-place bonding. Appl. Phys. Lett..

[16] Song E., Guo Q., Huang G., Jia B., Mei Y. (2017). Bendable Photodetector on Fibers Wrapped with Flexible Ultrathin Single Crystalline Silicon Nanomembranes. ACS Appl. Mater. Interfaces.

[17] Guo Q., Fang Y., Zhang M., Huang G., Chu P.K., Mei Y., Di Z., Wang X. (2017). Wrinkled Single-Crystalline Germanium Nanomembranes for Stretchable Photodetectors. IEEE Trans. Electron Devices.

[18] Roberts M.M., Klein L.J., Savage D.E., Slinker K.A., Friesen M., Celler G., Eriksson M.A., Lagally M.G. (2006). Elastically relaxed free-standing strained-silicon nanomembranes. Nat. Mater..

[19] Song E., Fang H., Jin X., Zhao J., Jiang C., Yu K.J., Zhong Y., Xu D., Li J., Fang G. (2017). Thin, Transferred Layers of Silicon Dioxide and Silicon Nitride as Water and Ion Barriers for Implantable Flexible Electronic Systems. Adv. Electron. Mater..

[20] Meitl M.A., Zhu Z.T., Kumar V., Lee K.J., Feng X., Huang Y.Y., Adesida I., Nuzzo R.G., Rogers J.A. (2006). Transfer printing by kinetic control of adhesion to an elastomeric stamp. Nat. Mater..

[21] Carlson A., Bowen A.M., Huang Y., Nuzzo R.G., Rogers J.A. (2012). Transfer printing techniques for materials assembly and micro/nanodevice fabrication. Adv. Mater..

[22] Menard E., Lee K.J., Khang D.Y., Nuzzo R.G., Rogers J.A. (2004). A printable form of silicon for high performance thin film transistors on plastic substrates. Appl. Phys. Lett..

[23] Stefanov P., Shipochka M., Stefchev P., Raicheva Z., Lazarova V., Spassov L. (2008). XPS characterization of TiO_2_ layers deposited on quartz plates. J. Phys. Conf. Ser..

[24] Zhang Y., Liu Y., Han G., Liu H., Hao Y. (2018). Improving metal/n-Ge ohmic contact by inserting TiO2 deposited by PEALD. Micro Nano Lett..

[25] Hobbs C., Fonseca L., Dhandapani V., Samavedam S., Taylor B., Grant J., Dip L., Triyoso D., Hegde R., Gilmer D. Fermi level pinning at the polySi/metal oxide interface. Proceedings of the 2003 Symposium on VLSI Technology. Digest of Technical Papers (IEEE Cat. No.03CH37407).

[26] Rim Y.S., Yang Y., Bae S.H., Chen H., Li C., Goorsky M.S., Yang Y. (2015). Ultrahigh and Broad Spectral Photodetectivity of an Organic-Inorganic Hybrid Phototransistor for Flexible Electronics. Adv. Mater..

